# Mitigating the adverse effects of urban environmental stress on individuals through piano performance with a natural theme: a case study from biophilia to soundscape healing

**DOI:** 10.3389/fpsyg.2026.1871520

**Published:** 2026-07-28

**Authors:** Tianyue Xing, Bo Zhao, Zhibo Chen

**Affiliations:** 1 School of Music, Nanjing Normal University, Nanjing, China; 2 China Communications Construction Group, Jiangsu Southern (Wuxi) Urban Construction Co. Ltd., Wuxi, China

**Keywords:** air pollution, heart rate, noise level, physiological responses, piano performance

## Abstract

Urban traffic-related air pollution and noise not only exert direct adverse effects on human health but also influence how individuals perceive and respond to these environmental stressors. Low-intervention strategies (e.g., outdoor music) have attracted increasing attention for their potential to alleviate physiological responses and discomfort induced by traffic-emitted pollution. The present study focuses on the mitigating effects of outdoor piano performance on the physiological responses of individuals exposed to traffic-related air pollution and noise. Field measurements are conducted in an outdoor cafe area located on a pedestrian street adjacent to a traffic roadway. Physical and physiological parameters are recorded over two comparative test days (with and without piano performance), and a subjective questionnaire survey is also administered. A total of 15 volunteers were initially recruited for the study. These participants were healthy male adults aged between 25 and 30 years, with heights ranging from 165 cm to 175 cm, and without any major medical conditions. The measured results indicate that air pollution and noise induced by road traffic have obviously affected human psychological reactions, and the relative *HR/SCL* indices increase with air pollutant concentrations (NO_2_/PM_10_) and noise levels. The presence of outdoor piano performance is capable of mitigating physiological reactions caused by traffic emissions, and the relative *HR*/*SCL* indices can be reduced by up to 50%. Traffic noise is clearly perceived by all volunteers and elicited varying degrees of dissatisfaction. The relevant statistical significance tests were conducted to compare the conditions with and without piano performance. Significant inter-departmental differences were found in *HR/SCL* indices (Wilcoxon rank-sum test) for the scenarios with and without piano performance. These results indicate that outdoor piano performance significantly influences human physiological responses and can effectively mitigate the adverse effects of traffic noise and air pollution on participants.

## 1 Introduction

Urbanization has led to increasingly complex ecological stressors, including intensified traffic flows, the resulting air pollution and noise, as well as multi-media environmental contamination involving air, water, and soil (Kabisch et al., 2017). Such environmental stressors exert wide-ranging impacts on human physical and mental health, contributing to the rising incidence of chronic and non-communicable diseases, including cardiovascular disorders (e.g., ischemic heart disease), lung cancer, asthma, and respiratory infections (Cai et al., 2022; Li et al., 2023; Liu et al., 2021; Zhu and Zhao, 2021). In addition, urban air pollution and noise can stimulate the autonomic nervous system, elevating physiological arousal levels (reflected in indicators such as heart rate and electrodermal activity) and thereby exacerbating discomfort and distress. This process may trigger a range of adverse emotional and physiological responses, which, over prolonged exposure, can further impair overall health and well-being (Chu et al., 2026; Higuera-Trujillo et al., 2021). Existing studies suggest that the integrated effects of urban environmental stressors on individuals are not solely determined by the levels of pollution and noise, but are also influenced by the interactions between environmental stressors (e.g., air pollution and noise) and the physiological stress responses of the human nervous system (Foellmer and Tyler, 2025).

Existing traffic management policies and engineering-based interventions (e.g., installation of outdoor air purifiers, noise barriers, and urban greening facilities) can effectively reduce human exposure to traffic-related emissions; however, they are often associated with high implementation costs and are not feasible in many contexts (Boppana et al., 2019; Li et al., 2023; Reiminger et al., 2020). Outdoor air purifiers have a limited spatial coverage, and large-scale deployment is required to address mobile urban traffic pollution sources or extensive target control areas, resulting in substantial capital investment and high operation and maintenance costs (Li et al., 2023). Noise barriers provide effective sound insulation and pollutant blocking performance, but they are primarily suitable for highways, with limited applicability in urban road environments dominated by mixed traffic conditions (Reiminger et al., 2020). Urban green infrastructure (e.g., street trees and shrubs) can simultaneously contribute to air pollution mitigation and noise attenuation over relatively large areas; nevertheless, their pollutant removal efficiency and noise reduction capacity remain limited, and their overall effectiveness in mitigating traffic emissions in real-world engineering applications is often marginal (Boppana et al., 2019).

Compared with strategies that directly reduce traffic emissions or personal exposure levels, a range of low-intervention approaches (such as fragrance diffusion, music exposure, and the release of negative air ions) do not directly reduce pollutant concentrations or noise levels. However, they may alleviate the stimulation of the autonomic nervous system induced by urban air pollution and noise, thereby reducing negative emotional and psychological responses and mitigating the adverse health effects associated with environmental pollution stressors (Ba and Kang, 2019b; Dansereau et al., 2026; Pino and Ragione, 2013; Zhang et al., 2026; Zhou et al., 2026). Previous studies have shown that, under relatively low noise conditions, rose fragrance can improve perceived audiovisual quality of traffic environments (Jiang et al., 2016). Similarly, lilac scent has been reported to enhance auditory comfort and reduce perceived traffic noise annoyance in urban street settings (Ba and Kang, 2019a).

Existing literature has also emphasized the important mechanisms by which beneficial soundscapes (e.g., musical soundscapes) contribute to stress recovery in humans (Zhou et al., 2026). In recent years, research has systematically revealed the multidimensional effects of piano playing on psychological or physiological regulation: actively playing or listening to piano music can effectively reduce physiological stress indicators such as cortisol levels and heart rate variability, significantly improve symptoms of depression and anxiety, and promote neuroplasticity by enhancing brain functional connectivity (Seinfeld et al., 2013; Toyoshima et al., 2011; Worschech et al., 2025). The relevant studies (Finnerty and Trainor, 2025; Salihu et al., 2024; Saskovets et al., 2025) have primarily focused on the capacity of piano performance to alleviate the adverse psychological effects induced by internal stressors, which are typically associated with individual psychological states or general daily stress. However, little attention has been paid to its potential mitigating effects on psychological burdens arising from external environmental stressors, such as air pollution and traffic noise. Furthermore, previous research has not systematically quantified the extent to which piano performance modulates autonomic nervous system activity, as reflected by physiological indicators including heart rate (*HR*) and skin conductance level (*SCL*). Nor has it thoroughly elucidated the nonlinear quantitative relationships between these physiological responses and environmental exposure variables, including concentrations of air pollutants (NO_2_ and PM_10_) and ambient noise levels.

The present research was conducted in an outdoor cafe area within a pedestrian street adjacent to a traffic corridor. Physical and physiological parameters were measured across two comparative test days (i.e., with and without piano performance). In addition, subjective questionnaire surveys were administered. Based on these data, we further examined the quantitative relationships between physical environmental parameters and physiological responses, and evaluated the extent to which outdoor piano performance could mitigate negative emotions and physiological stress responses, particularly the autonomic nervous system activation induced by environment-related air pollution and noise exposure. The primary scientific contribution of this study lies in its investigation, through a combination of experimental measurements and subjective questionnaire surveys, of the mitigating effects of outdoor piano performance on the adverse physiological impacts induced by traffic noise and air pollution. Specifically, the study evaluated these effects using physiological indicators, including heart rate (*HR*) and skin conductance level (*SCL*). Although outdoor piano performance cannot reduce individuals’ actual exposure to traffic-related air pollution and noise, it can alleviate the physiological responses and discomfort induced by such emissions. As such, it represents a low-intervention strategy that is beneficial to human physical and mental well-being.

## 2 Methodology

In the methodology part, the research site, physical parameters measurement, physiological parameters measurement, and operational procedure are described in detail. The influences of piano performance on physiological reaction of human can be analyzed and revealed based on the experimentally measured data described above.

### 2.1 The information of target site

**Figure 1:**
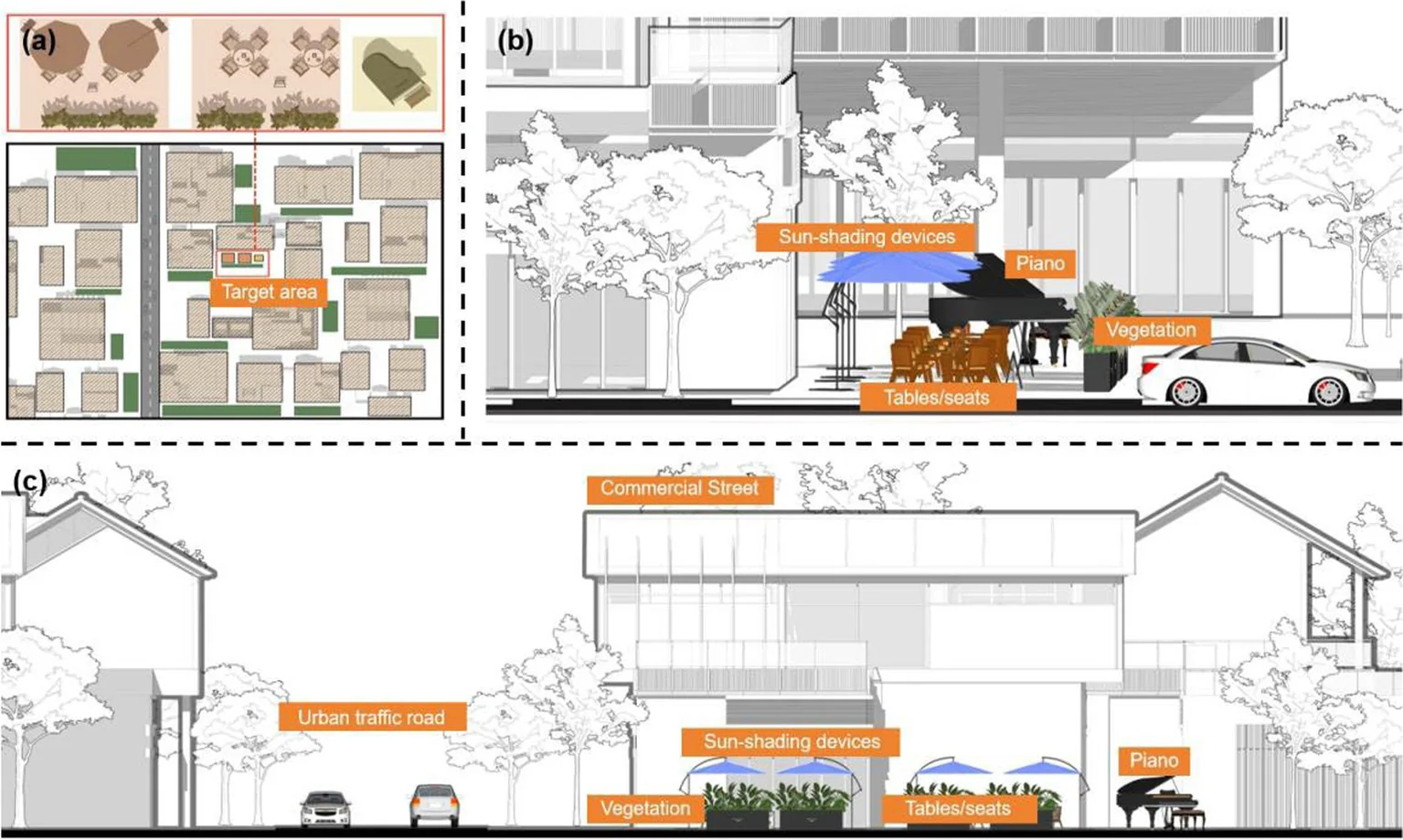
The information of targeted research area: outdoor cafe area.

The target scenario investigated in this study (an outdoor cafe area) is located in Nanjing, Jiangsu Province, China. The outdoor cafe area is situated within an urban pedestrian district, in close proximity to a motor vehicle roadway at an approximate distance of 18 m, offering the advantage of convenient accessibility. As illustrated in Figure 1, the outdoor cafe area is equipped with tables and chairs, piano and landscaped greenery from nearby vehicular environment generate both air pollution and noise, posing exposure risks to individuals within the cafe environment and potentially inducing corresponding physiological and physiological responses. To mitigate these adverse effects, this study utilized piano performances to generate soothing and esthetically pleasing music, thereby alleviating the negative impacts caused by environmental emissions (including air pollutants and noise).

The researchers selected Schumann’s “Waldszenen” (Op. 82) as the experimental music. Comprising nine short, exquisite piano pieces, this work depicts the ever-changing scenery of the forest with a Romantic touch. Through flexible rhythmic breathing, dissonances and tonal shifts, as well as intricate polyphonic textures, the music achieves a poetic interpretation of the forest as a “community of life.”As listeners immerse themselves in this music, they are unconsciously transported to a mental state reminiscent of being in a forest: melodies that sound like birdsong, rhythms that evoke the swaying of tree shadows, and the occasional blast of a hunting horn—all of which awaken an innate longing for the harmony of nature while bringing a sense of tranquility, reverie, and even a touch of mysterious security. This experience not only alleviates the mental fatigue of urban life but also, through an auditory journey that “simulates nature,” evokes the same sense of relaxation and belonging one feels when actually in a forest. In addition, a complete set of tables and chairs located at a corner of the outdoor cafe area was designated as the experimental station. This space was used for the installation of various sensors and cameras, as well as to provide resting and on-site data processing space for the participants.

### 2.2 The physical parameters measurement

In the current study, the targeted physical parameters include road traffic flux, traffic-emitted air pollutant concentrations, air temperature and humidity, and traffic noise level. Owing to the presence of effective shading facilities, the influence of solar radiation on occupants’ psychological and physiological responses was not considered in this study,the noise levels generated by motor vehicles on the adjacent roadway were recorded. Apart from traffic noise, no other significant noise sources were identified in the study area.

**Figure 2:**
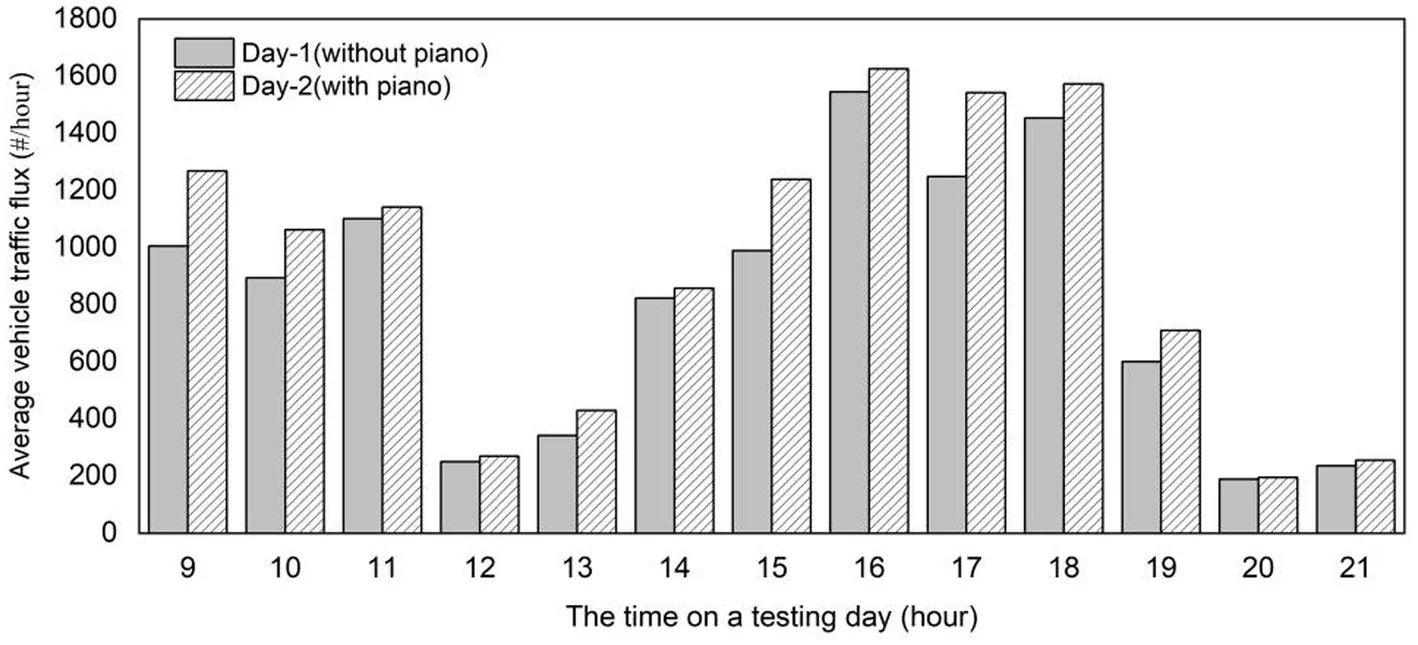
The measured traffic fluxes in the two typical days.

Due to the traffic regulations implemented in urban central districts, the presence of heavy-duty vehicles is negligible, while light-duty vehicles constitute the dominant mode of transportation (Chang and Hsiao, 2026). The traffic flow of light-duty vehicles along the urban roadway within this historic district was quantified through the analysis of camera recordings. The temporal variations in traffic flow during peak and off-peak periods are illustrated in Figure 2 and are further elaborated in the subsequent section. In this study, the defined peak periods (07:00–13:00 and 14:00–20:00) are longer than those typically observed on conventional urban roads outside the study area (Chang et al., 2022; Chang and Hsiao, 2026). This extension can be attributed to the combined effects of tourism-related mobility and routine commuting activities.

In the previous study, the main types of traffic-emitted air pollutants includes airborne particles and nitrogen oxides. Therefore, the concentrations of PM_10_ and NO_2_ are measured to reveal the severity of pollution. The TSI-8530 (DustTrak) and TSI-8145 (BlueSky NO_2_ sensor) are utilized to measure the PM_10_ and NO_2_ concentrations (μg/m^3^), respectively. The sound level sensor (LoRaWAN) is adopted to measure the traffic noise level (dB) and quantitatively characterize the piano performance. The HOBO® Temp Data Loggers are directly used to measure the air temperature (°C) and humidity (%) in this outdoor cafe area. Regarding the long term measurement (about 10 h in a day) in the present study, the sampling period of meteorological parameters (air temperature and humidity), air pollutants (PM_10_ and NO_2_), and noise level are 1 min.

### 2.3 The physiological parameters measurement

Based on the previous study (Foellmer and Tyler, 2025), the HR (heart rate) and EDA (electrodermal activity) are measured to reveal the influences of traffic-emitted air pollutants and noise level on physiological reactions of humans sitting in the targeted outdoor cafe area. Heart rate (HR) denotes the mean number of cardiac beats recorded over a defined time interval. Electrodermal activity (EDA), regulated by sweat gland function, represents variations in the skin’s electrical conductivity in response to emotional or environmental stimuli.

Physiological arousal is elicited when the autonomic nervous system is activated in response to stimuli of emotional or physical salience. Environmental stressors, including noise and air pollution, may intensify physiological arousal, thereby exacerbating discomfort or unease and, over prolonged exposure, potentially impairing both physiological and psychological health. The assessment of physiological arousal offers critical insight into individuals’ implicit responses to complex multisensory environments, thereby complementing subjective dimensions of people-environment interactions obtained through self-reported measures. Such arousal is typically reflected in variations in heart rate, electrodermal activity, and other physiological processes regulated by the sympathetic nervous system.

Heart rate (HR) and electrodermal activity (EDA) are experimentally acquired using a wireless Shimmer3 GSR + unit (sampling frequency: 128 Hz) and logged via the ConsensysPRO software. The detailed information about the measurement devices described above can be referred to the existing literature (Foellmer and Tyler, 2025). The EDA signal consists of two main components: the skin conductance level (*SCL*) and the skin conductance response (*SCR*). This present study concentrates on skin conductance level (*SCL*), as tonic activity more effectively represents individuals’ overall arousal states in response to the integrated sensory environment, including the interplay between the ambient smellscape and soundscape. In contrast, phasic activity primarily reflects transient, stimulus-specific responses to discrete auditory or olfactory events.

The two dimensionless indices have been defined as the relative *HR* index (%) and relative *SCL* index (%), as shown by Equations 1, 2. The subscript of m and b represents the baseline and measured the heart rate and the skin conductance level, respectively. Previous studies have demonstrated that air pollution and environmental noise can significantly affect heart rate (*HR*, bpm) and skin conductance level (*SCL*, μS; Foellmer and Tyler, 2025). To facilitate comparisons of physiological responses among different individuals under the combined effects of air pollution, traffic noise, and piano performance, two dimensionless indicators, namely the *HR* index and *SCL* index, were adopted in this study, as defined in Equations 1, 2. The baseline values of *HR* and *SCL* were calculated as the averages measured during the preceding resting condition, a method widely adopted in previous studies (Foellmer and Tyler, 2025). In the present study, the baseline *HR* (*HR*_b_) and *SCL* (*SCL*_b_) values for Volunteer No. 1 were 65 bpm and 5.5 μS, respectively.


$$\mathrm{HR}-\text{index}=(HR_{b}-HR_{m})/HR_{b}$$ (1)

$$\mathrm{SCL}-\text{index}=(\mathrm{SC}L_{m}-\mathrm{SC}L_{b})/\mathrm{SC}L_{b}$$ (2)

Except for the physiological and psychological parameters described above, we also conducted subjective questionnaire survey to reveal whether the participants expressed dissatisfaction or complaints regarding traffic noise and air pollution. Regardless of the presence of piano performance, the volunteers were consistently exposed to both traffic noise and air pollution. However, vehicular exhaust was barely perceptible to the participants, resulting in negligible complaints regarding traffic-related air pollution. In contrast, traffic noise was clearly perceived by all volunteers and elicited varying degrees of dissatisfaction. For both the piano-intervention and non-intervention test days, participants’ complaints about traffic noise were recorded hourly, specifically whether they experienced discomfort caused by traffic noise. The traffic noise complaint percentage/ratio was defined as the ratio of the number of participants reporting discomfort to the total number of participants.

### 2.4 The operational procedure of experimental measurement

This study conducted field experiments over a two-week period in April 2024, representing spring conditions, with a total of 11 valid measurement days, all under clear weather. The daytime air temperature was approximately 20 °C, with relative humidity around 50%, indicating generally comfortable outdoor thermal and humid levels. Experimental measurements under two conditions (without piano performance and with piano performance) were carried out on alternating days. Given that traffic-related air pollution and noise levels varied daily, two representative days with comparable pollution and noise conditions were ultimately selected for analysis, corresponding, respectively, to the no-music and music scenarios. Within the outdoor leisure area of the café, windbreak landscaping features and shielding panels were incorporated into the architectural design. As a result, the local wind velocity remained below 0.3 m/s, thereby minimizing the potential adverse effects of excessive outdoor wind on the physiological responses of occupants.

Measurements were conducted only on clear days; experiments were postponed during overcast or rainy conditions and resumed on the next suitable day. A total of 15 volunteers participated in this study. Each measurement day started at 09:00 and ended at 21:00. During the experimental period, physical environmental parameters were continuously monitored using various sensors, whereas physiological responses were assessed intermittently to ensure sufficient rest for participants. Within each hourly cycle, participants underwent physiological measurements during the first 30 min, followed by a 30-min rest period, during which they were allowed to leave the outdoor cafe area. During testing periods, participants were permitted to use laptops or smartphones.

### 2.5 Detailed information on participants and volunteers

A total of 15 volunteers were initially recruited for the study; however, due to personal constraints and the demanding nature of the experimental protocol, only three participants completed the full set of measurements. These participants were healthy male adults aged between 25 and 30 years, with heights ranging from 165 cm to 175 cm, and without any major medical conditions. Based on the health status of the three participants (e.g., absence of illness such as colds) and the consistency of their physiological responses, data from one male participant were ultimately selected for detailed analysis. Throughout each test day, all 15 volunteers maintained stable emotional states, and no major life or work-related events occurred that could potentially influence their physiological responses. In addition, participants were prohibited from consuming caffeine during the experimental period, and individuals with caffeine dependence were excluded from participation in this study.

### 2.6 Data processing and statistical analysis

Data processing was conducted using MATLAB and Origin software, including the calculation of descriptive statistics such as means and standard deviations. Specifically, this present study characterized the physiological parameters (*HR/SCL* indexes) in the two conditions with and without the piano performance, compared inter-departmental differences using the Wilcoxon rank-sum test [1], and assessed the practical role of piano performance in mitigating the negative impacts of air pollution and traffic noise on volunteers.

## 3 Results and analysis

In the results part, the experimentally measured physical (section-(3.1)) and physiological (section-(3.2)) parameters are described firstly. And then the quantitative relationship between the physical and physiological parameters is analyzed in section-(3.3). In the following parts, the “Day-1” and “Day-2” refers to the testing days without and with piano performance. The traffic flux, air pollutant concentration, and noise level are similar. Hence, these 2 days can be used as testing days to investigate the influence of piano performance on physiological reactions of humans.

### 3.1 The experimental results of physical parameters

Figure 2 shows the measured traffic vehicle fluxes in the two typical days with and without piano performance. Different from other physical sensors (e.g., air pollutants, temperature/humidity) placed in the targeted cafe area, the camera is installed near the road for vehicle recording. These two typical days are weekdays, and the commuter transportation is dominant. The peak period is 9:00–12:00 (AM) and 16:00–19:00 (PM). The vehicle fluxes in these 2 days are quite similar, and the relative differences range from 3 to 20%. From the perspectives of the similar traffic fluxes, these 2 days can be used as testing days for comparison.

**Figure 3:**
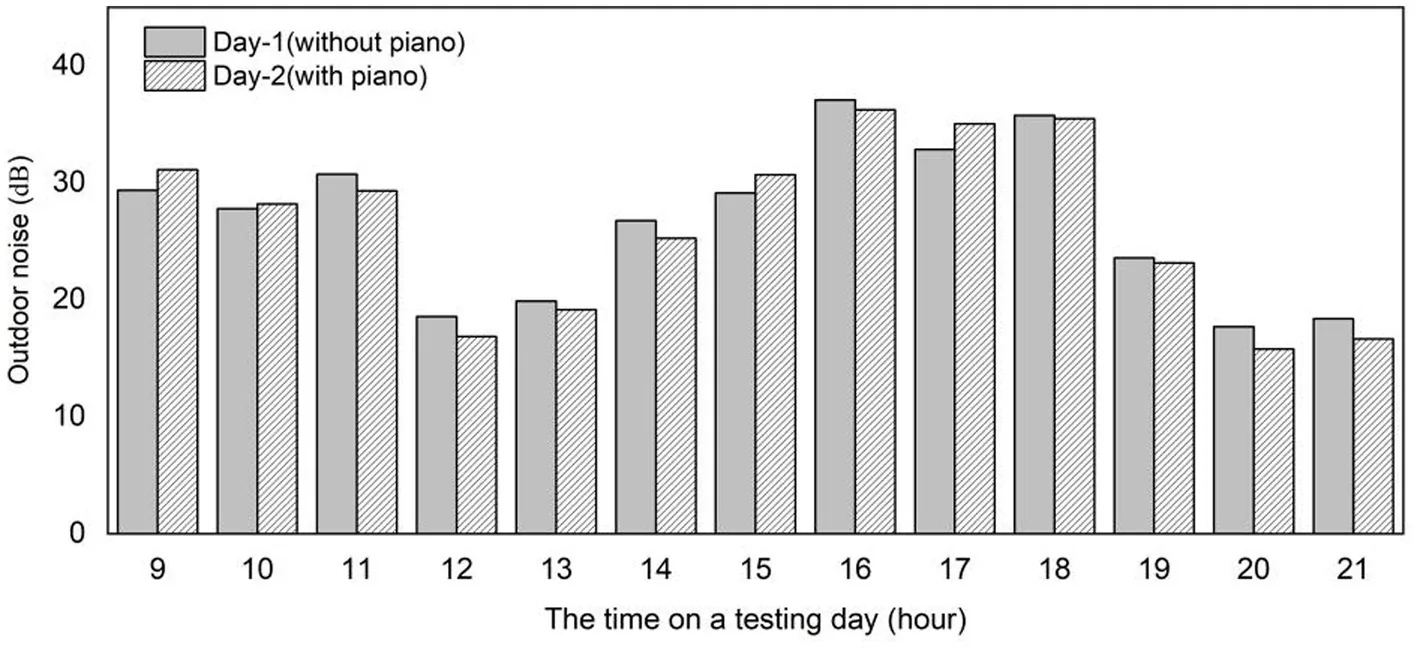
The measured noise level in the two typical days.

Figure 3 shows the measured noise level in the two typical days with and without piano performance. The main noise source is road traffic due to the weekdays, and the surrounding environment of the targeted outdoor cafe area is relatively quiet. For the traffic peak hours, the noise level is relatively higher than those of the off-peak hours. The noise level in these 2 days are quite similar, and the relative differences range from 1.0 to 12%. Therefore, the comparable noise levels observed on the two testing days provide a reliable basis for examining the effects of piano performance on physiological responses.

**Figure 4:**
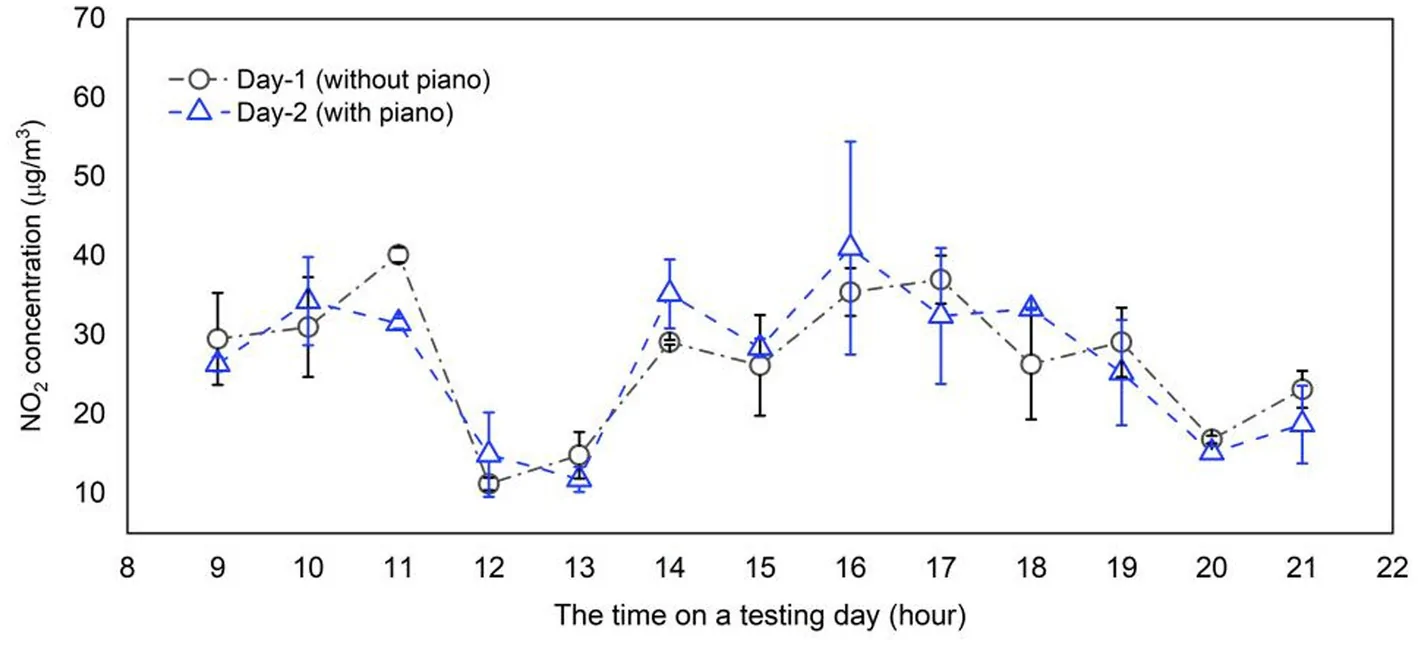
The measured NO_2_ concentrations in the two typical days.

Figure 4 shows the measured NO_2_ concentrations in the two typical days with and without piano performance. The previous literature indicates that is one of the main traffic-emitted airborne gaseous pollutants (Azhari et al., 2018). Hence, the NO_2_ concentrations are measured to characterize the severity of air pollution. During the off-peak period (12:00–13:00; 20:00–21:00), the NO_2_ concentrations are obviously lower, resulting from the lower road vehicle fluxes. The maximum NO_2_ concentrations occur at 11:00 (AM) and 16:00 (PM). The NO_2_ concentrations in these 2 days are similar, and the relative differences range from 7 to 25%. As indicated by previous literature (Azhari et al., 2018), the NO_2_ concentrations higher than 30 μg/m^3^ have obvious effects on building material corrosion. Hence, the NO_2_ concentrations level (shown in Figure 4) may negatively affect human health.

**Figure 5:**
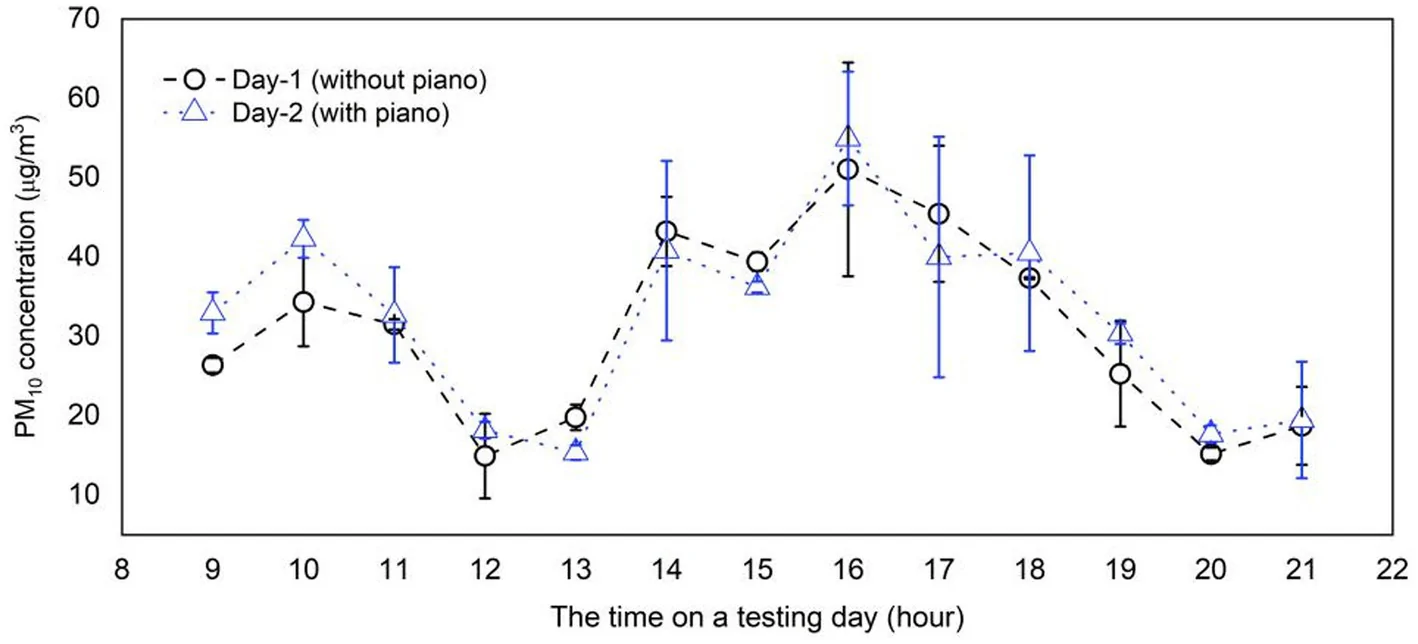
The measured PM10 concentrations in the two typical days.

Figure 5 shows the measured PM_10_ concentrations in the two typical days with and without piano performance. The variation trend of PM_10_ is similar to that of NO_2_, indicating that the road traffic is the main source of air pollution in weekdays. The maximum airborne PM_10_ concentrations are observed at 11:00 (AM) and 16:00 (PM). The relative differences of PM_10_ concentrations in these 2 days range from 4 to 21%. During the weekdays, surrounding environment of the targeted cafe area is relatively clean, due to the low pedestrian volume in this pedestrianized street. Although air pollution at this concentration range may not be perceptible to individuals, PM_10_ can still exert adverse effects on physiological responses and human health.

### 3.2 The experimental results of physiological parameters

**Figure 6:**
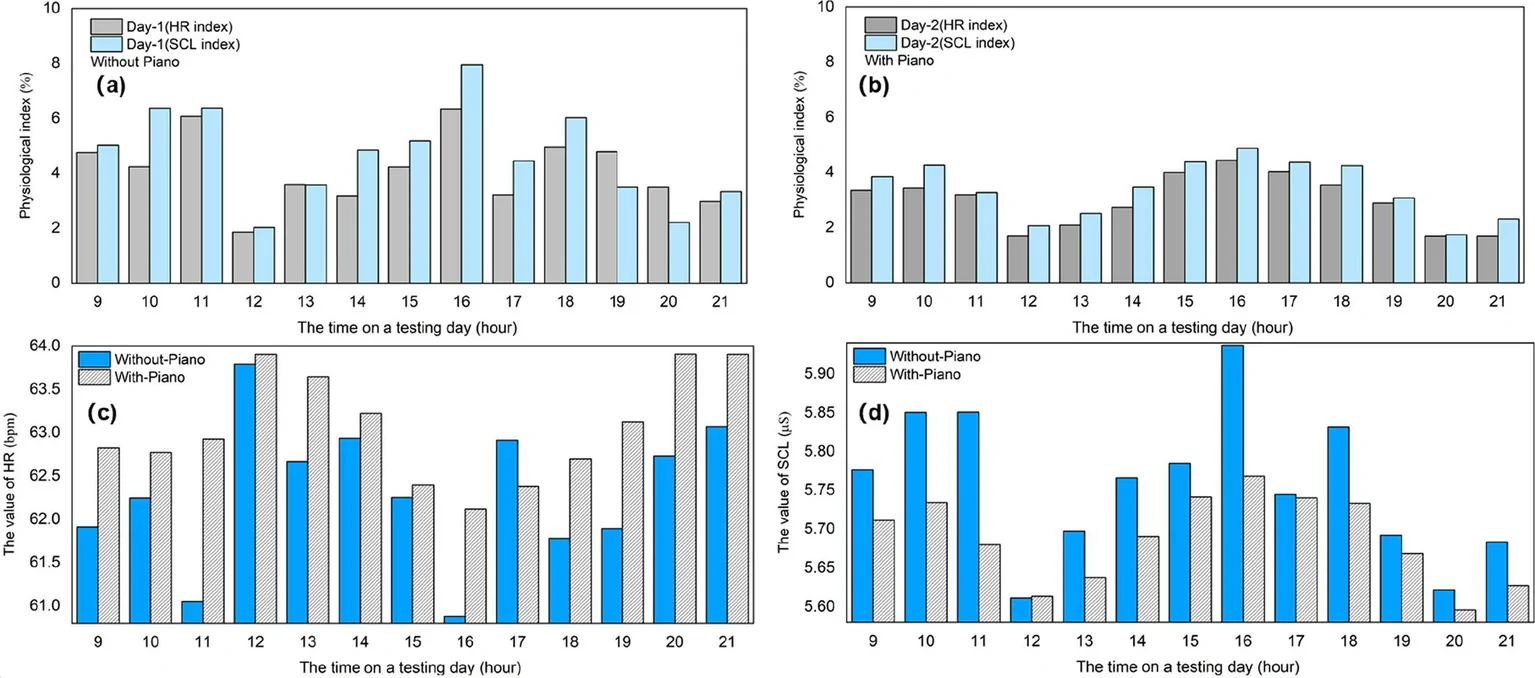
The measured HR/SCL on the two testing days: **(a)** Relative index on the Day-1 without piano performance; **(b)** Relative index on the Day-2 with piano performance; **(c)** The actual value of HR on the two testing days; **(d)** The actual value of SCL on the two testing days.

Figure 6 describes the measured actual values and relative indexes of *HR* and *SCL* for the Volunteer-1, who have completed the full set of measurements. Figure 6a shows the measured relative *HR* index and *SCL* index in the testing day without piano performance (Day-1). The higher *HR*/*SCL* indexes indicate that individuals are subjected to more pronounced and severe impacts from traffic-related pollution and noise. During the traffic peak period (9:00–11:00; 15:00–16:00; 17:00–19:00), the *HR*/*SCL* indexes are obviously higher than those at other time periods. The relative *HR*/*SCL* indexes in the present study are higher than those in the existing literature (Kabisch et al., 2017), and the influence of urban road traffic in practical conditions (up to 6%) may be more obvious than the laboratory measurements (1–4%). As described by Figure 6b, the *HR*/*SCL* indexes are reduced once the piano performance is adopted. The esthetic and soothing piano performance can significantly alleviate individuals’ physiological responses induced by environmental stressors such as air pollution and noise. Similar to the Day-1 without piano performance, the *HR*/*SCL* indexes in off-peak hours (12:00–13:00 and 20:00–21:00) of Day-2 are lower. Piano performance cannot fully eliminate individuals’ physiological responses induced by environmental stressors such as pollution; even under the piano-music condition, varying pollution concentrations and noise levels continue to exert differential effects on individuals. Figures 6c,d shows the actual values of *HR* (bpm) and *SCL* (μS), and the relative indexes can be obtained based on the experimentally measured actual values and Equations 1, 2.

**Figure 7:**
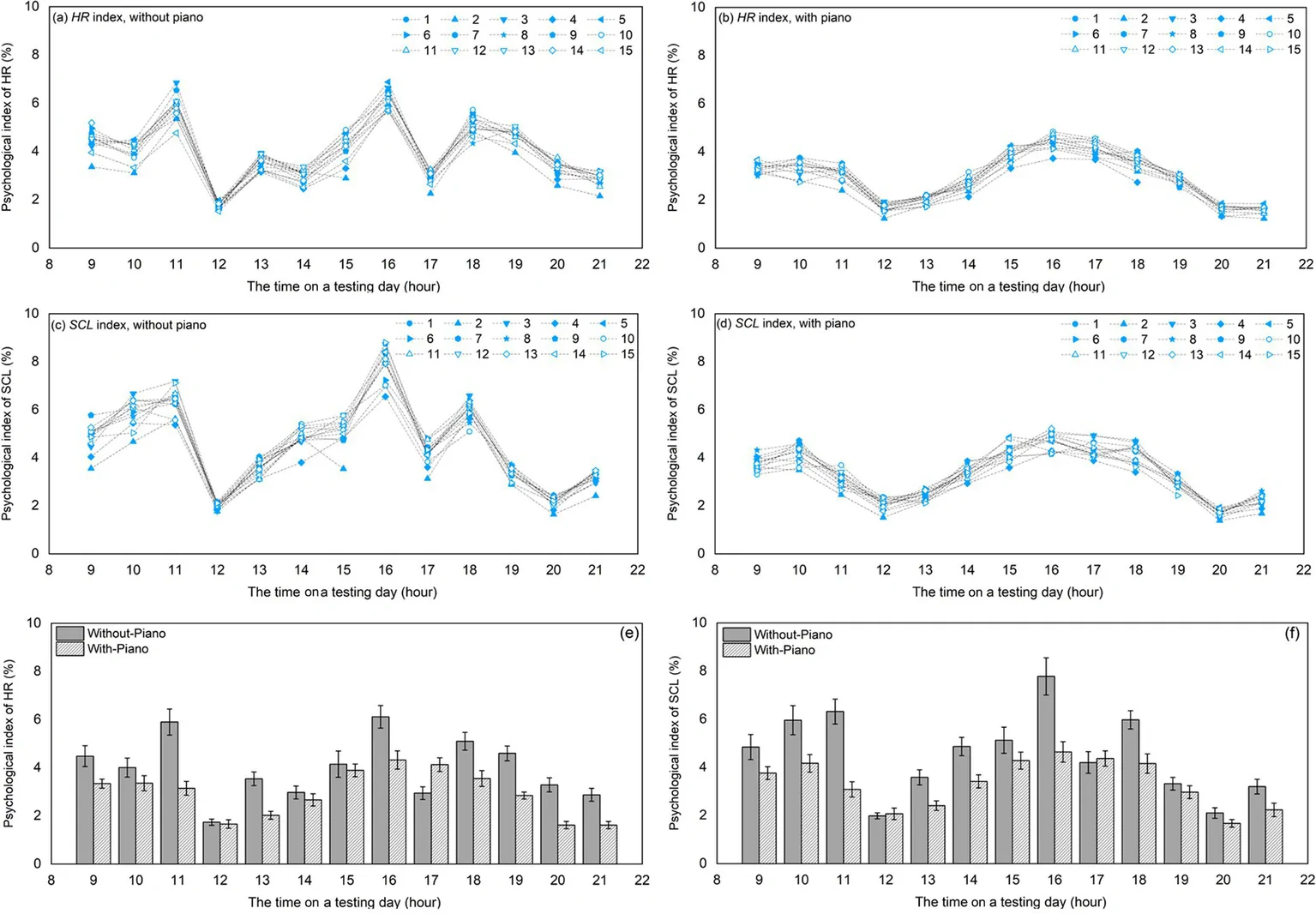
The measured HR/SCL indexes for all of the volunteers: **(a)** The HR index on the Day-1 without piano performance; **(b)** The HR index on the Day-2 with piano performance; **(c)** The SCL index on the Day-1 without piano performance; **(d)** The SCL index on the Day-2 with piano performance; **(e)** The average HR index for all of the fifteen volunteers; **(f)** The average SCL index for all of the fifteen volunteers.

Figures 7a,b present the *HR* index measurements of the 15 volunteers under the conditions with and without piano performance, respectively. The temporal variation patterns of the *HR* index were generally consistent across volunteers throughout the day, while the magnitude of *HR* index fluctuations at specific time points could exceed 1%. Complete datasets were obtained from Volunteers No.1–3, whereas the remaining 12 volunteers each missed 1–4 measurements. Despite these limited missing data, the dataset remains a reliable basis for quantitative analysis. Figures 7c,d illustrate the *SCL* index measurements. The temporal trends of the *SCL* index were similar to those observed for the *HR* index; however, the inter-individual variability in *SCL* index values at the same time point was greater than that of the *HR* index. Figures 7e,f show the mean values and standard deviations of the *HR* index and *SCL* index, respectively, for all 15 volunteers. Based on the data presented in Figures 7e,f, statistical significance tests were conducted to compare the conditions with and without piano performance. The Wilcoxon test yielded *p*-values of 0.003418 and 0.001221 (*p* < 0.01) for the *HR* index and *SCL* index, respectively. The paired-sample analyses demonstrated that both *HR* and *SCL* indices under the piano-performance condition were significantly lower than those measured under the corresponding no-piano condition. Following the implementation of the piano-performance intervention, the mean *HR* index decreased by 1.0420, corresponding to a reduction of 26.19%. The paired t-test yielded a statistically significant result, t(12) = 3.7392, *p* = 0.002826. Similarly, the mean *SCL* index decreased by 1.2334, representing a reduction of 27.06%, with the paired t-test indicating a significant difference, t(12) = 4.1605, *p* = 0.001322. Consistent with the paired t-test results, the Wilcoxon signed-rank test also revealed significant differences between the two conditions, confirming the robustness of the observed effects. These results indicate that outdoor piano performance significantly influences human physiological responses and can effectively mitigate the adverse effects of traffic noise and air pollution on occupants.

**Figure 8:**
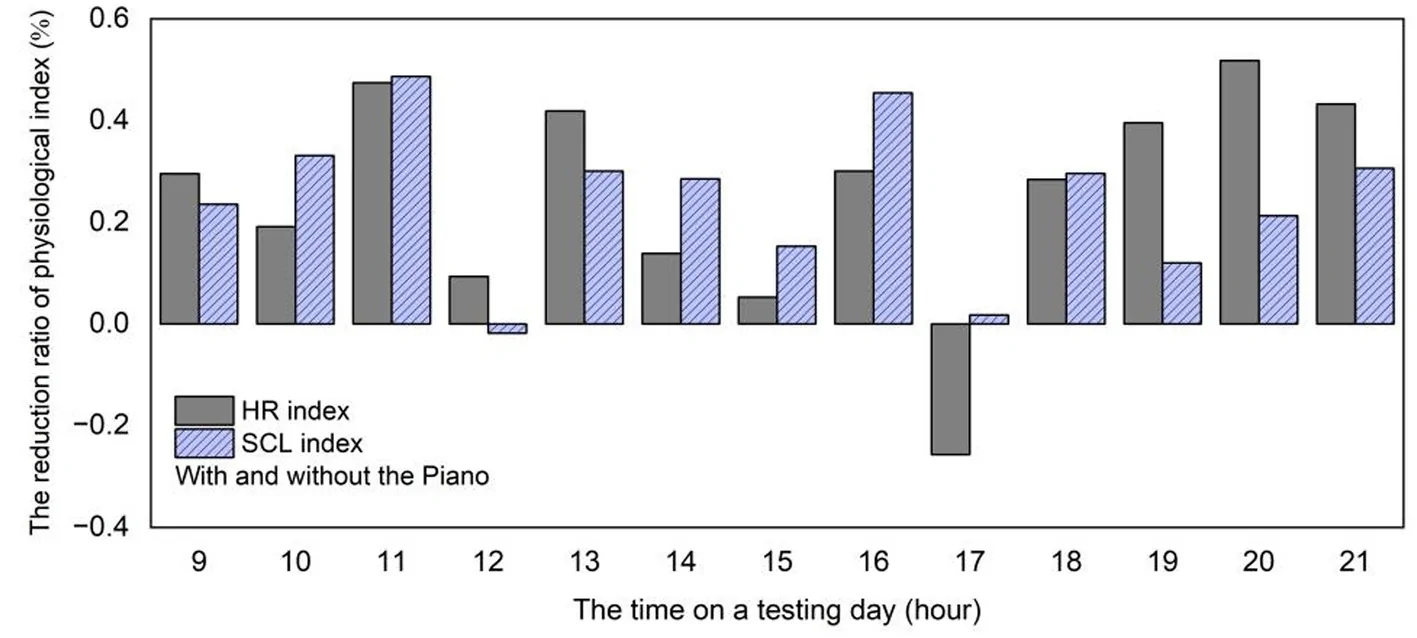
The reduction ratio of the two physiological parameters by adopting piano performance.

Figure 8 describes the reduction ratio of the two physiological parameters (the relative *HR* and *SCL* indices). The Equation 3 is utilized to calculate the reduction ratio. A higher value of the reduction ratio indicates a more pronounced soothing/mitigation effect of the piano performance. The maximum reduction ratio of the *HR* and *SCL* indices are approximately 0.5, and the average *HR* and *SCL* indices are 0.1 and 0.2, respectively. At 17:00 (PM), the reduction ratio of *HR* index is negative, indicating that the piano performance poses negative effects on physiological reactions. This phenomenon may be caused by experimental errors or personal emotion variations. Overall, the piano performance has obvious mitigation effects on physiological reactions, which is caused by the traffic-emitted air pollutants and noise.


$$\text{reduction}-\text{ratio}=(X_{\mathrm{wp}}-X_{p})/X_{\mathrm{wp}}$$ (3)

Where *X* represents the relative *HR* and *SCL* indices, and the subscripts of “*wp*” and “*p*” represent the experimental conditions with and without the piano performance.

### 3.3 The relationship between physical and physiological parameters

**Figure 9:**
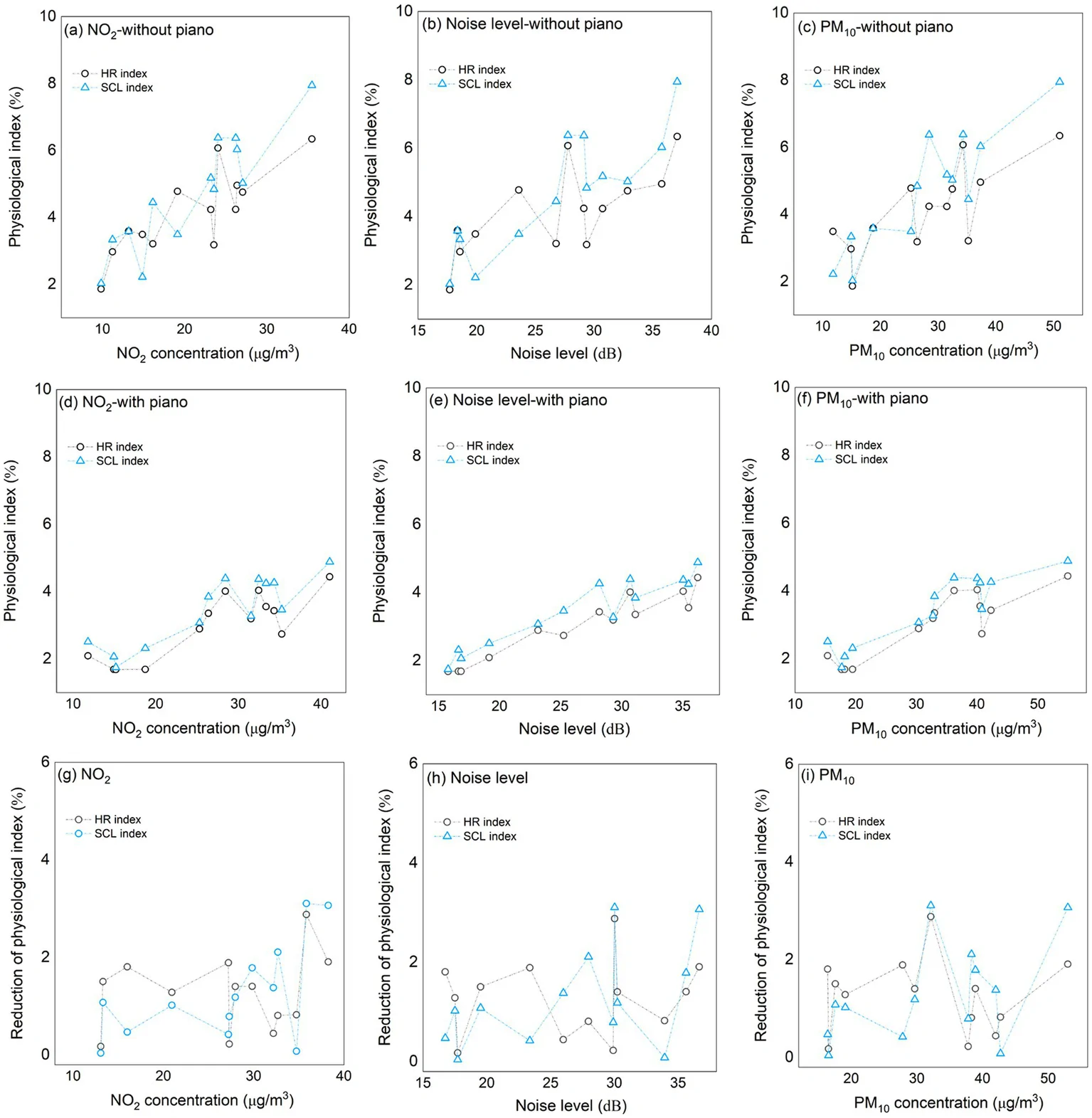
The relationship between physical and physiological parameters: **(a)** HR/SCL indices, NO_2_, without piano; **(b)** HR/SCL indices, noise, without piano; **(c)** HR/SCL indices, PM_10_, without piano; **(d)** HR/SCL indices, NO_2_, with piano; **(e)** HR/SCL indices, noise, with piano; **(f)** HR/SCL indices, PM_10_, with piano; **(g)** The reduction of HR/SCL indices, NO_2_, with piano; **(h)** The reduction of HR/SCL indices, noise, with piano; **(i)** The reduction of HR/SCL indices, PM_10_, with piano.

Figures 9a–c illustrates the quantitative relationship between the experimentally measured physical and physiological parameters on the testing day without piano performance (Day-1). The physical parameters include NO_2_ concentrations, noise level, and PM_10_ concentrations, and the physiological parameters are the measured relative *HR* index and *SCL* index. As shown in Figure 9, the range of physiological parameters is 1.5–8.0%, while the ranges of physical parameters for NO_2_, noise, and PM_10_ are 10–40 μg/m^3^, 15–40 dB, and 10–55 μg/m^3^, respectively. Higher air pollutant concentrations and noise levels lead to stronger physiological reactions and relative *HR*/SCL indices. The positive correlation between the physical and physiological parameters is observed. The influences of NO_2_ and PM_10_ concentrations on physiological reactions are similar. Figures 9d–f describes the influences of air pollutant concentration and traffic noise level on *HR* and *SCL* indices on the testing day with piano performance (Day-2). As shown in Figure 9d, when the NO_2_ concentration increases from about 10 μg/m^3^ to 40 μg/m^3^, the *HR* and *SCL* indices increase approximately by 2%, and a similar trend can be observed in the results of PM_10_. When the traffic noise increases from 15 dB to 37 dB, the *HR* index varies from lower than 2% to higher than 4%, and the increase in the *SCL* index is greater than that in the *HR* index. Piano performance intervention significantly reduced both the *HR* index and *SCL* indices. Meanwhile, the *HR* and *SCL* indices still exhibited significant positive correlations with air pollution concentration and traffic noise level. Figures 9g–i illustrates the effects of different air pollution concentrations and traffic noise levels on the reductions in the *HR* and *SCL* indices. The reduction in the *HR/SCL* indices was defined as the difference between the values measured before and after the piano performance intervention. A larger reduction indicates a stronger intervention effect of piano performance on physiological responses. As shown in Figures 9g–i, neither air pollution concentration nor traffic noise level exhibited a clear trend in relation to the reductions in the *HR* and *SCL* indices (e.g., positive or negative correlations). Future studies will collect a larger experimental dataset to further elucidate the underlying mechanisms through which air pollution concentration and traffic noise level influence the reductions in the *HR/SCL* indices induced by piano performance intervention.

## 4 Discussion

In this part, the results of subjective questionnaire survey are discussed. And then the research limitations are listed and analyzed.

### 4.1 The results of subjective questionnaire survey

**Figure 10:**
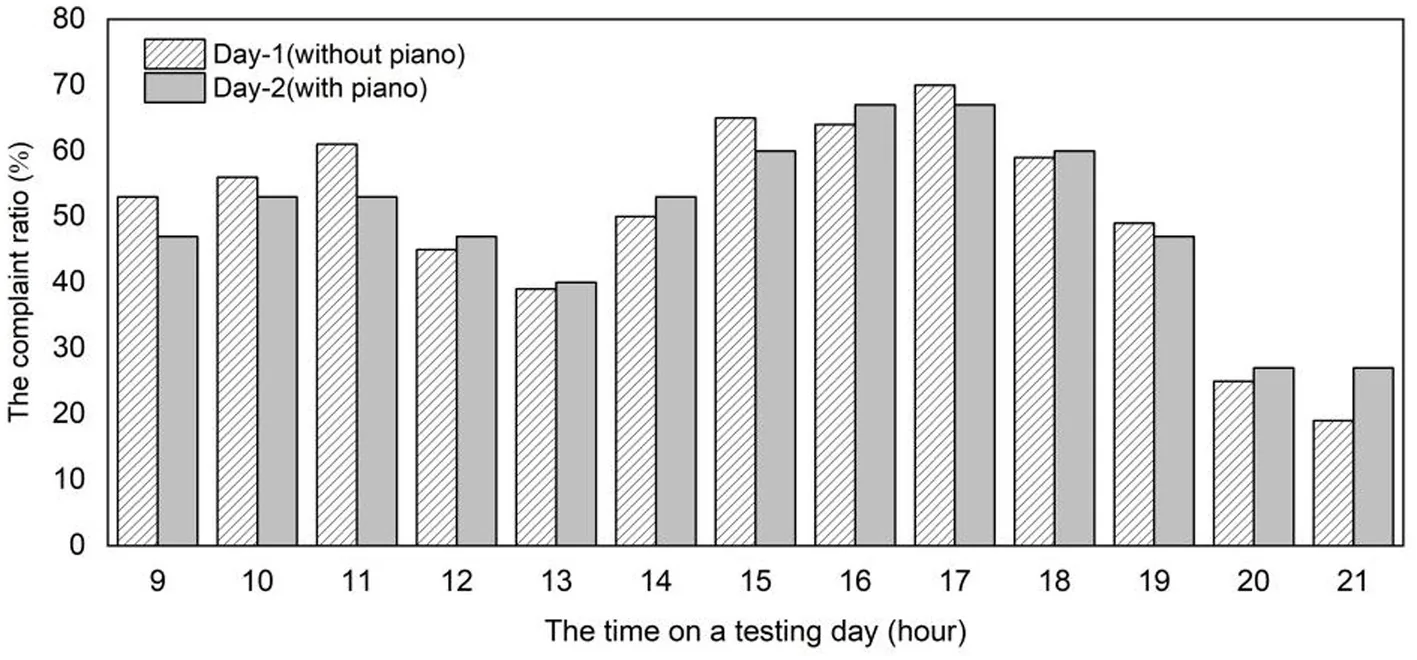
The results of subjective questionnaire survey: Complaint ratio of traffic noise.

As illustrated in Figure 10, the complaint ratio is defined as the proportion of volunteers reporting noise-related complaints relative to the total number of respondents. As shown by Figure 10, the piano performance intervention exhibited only a weak and statistically insignificant effect in reducing the proportion of participants reporting annoyance from traffic noise, likely because the traffic noise remained perceptible to the participants throughout the intervention. Based on the results presented in Figure 10, the measured traffic noise complaint percentages collected at different times throughout the testing day were used to calculate the daily average traffic noise complaint ratio under both the piano-performance and non-piano-performance conditions. This daily average traffic noise complaint ratio is, 50.4 and 49.7% for the conditions without and with piano performance. The complaint ratio with slightly higher values is observed during peak traffic periods compared to off-peak times. Therefore, from the perspective of subjective perception, the mitigation of traffic noise appears to be of substantially greater importance than the control of traffic-related air pollution. In the subjective survey of traffic noise perception conducted in this study, only 15 volunteers were recruited. Future research will involve a substantially larger sample size to further investigate subjective responses and to more comprehensively elucidate the mechanisms by which outdoor piano performances influence human perception and evaluation of traffic noise pollution.

### 4.2 Comparison with the relevant literature

Compared with existing studies on piano-based and other music-related physiological interventions, the present study possesses stronger practical relevance and a more pronounced engineering-oriented perspective. Most previous investigations (e.g., Seinfeld et al., 2013; Toyoshima et al., 2011; Worschech et al., 2025) were conducted in laboratory or clinically controlled settings and primarily focused on the long-term effects of active piano training on cognitive function, emotional well-being, and quality of life, or on changes in stress-related hormones (e.g., cortisol) associated with different creative activities.

In recent years, research paradigms in this field have continued to expand. Finnerty and Trainor (2025) conducted a randomized controlled trial of group music therapy among university students and demonstrated its long-term stress-reduction benefits through hair cortisol measurements and psychometric assessments. Wei et al. (2025) combined music intervention with physical exercise and confirmed the persistence of intervention effects through follow-up questionnaire surveys. Salihu et al. (2024), through an umbrella review of 20 systematic reviews, verified the effectiveness of music interventions in alleviating anxiety among patients with dementia. Zhang (2025) and Yang (2025) further performed randomized controlled studies of piano-based music therapy for adolescents with anxiety disorders. Zhang reported that the therapeutic benefits were maintained throughout a one-year follow-up period, whereas Yang demonstrated a synergistic enhancement effect when piano melodies were combined with physical exercise interventions. In addition, Saskovets et al. (2025) systematically evaluated the effects of various sound-based interventions on reducing physiological stress biomarkers, including cortisol, heart rate variability, and blood pressure, through a scoping review.

Nevertheless, most existing studies continue to rely on randomized controlled experimental paradigms, with interventions predominantly implemented in therapy rooms, laboratories, or other highly controlled environments. Moreover, the stressors examined have largely been limited to internal physiological conditions or general everyday stress. Consequently, little attention has been paid to the potential mitigating effects of piano performance on the adverse physiological impacts induced by external environmental stressors, such as air pollution, traffic noise, and other forms of environmental burden. This knowledge gap highlights the necessity of investigating the role of piano performance as a practical environmental intervention strategy in real-world outdoor settings characterized by multiple concurrent environmental stressors.

In contrast, the present study innovatively employed piano music as a soundscape-based intervention within a real urban outdoor open space, directly addressing the combined environmental stressors of traffic-related air pollution and noise. To the best of our knowledge, this represents one of the few empirical studies that has evaluated the effectiveness of music-based soundscape interventions under uncontrolled urban exposure conditions. The findings not only demonstrate that live piano performance can substantially mitigate psychophysiological stress responses induced by environmental exposure, with reductions in the relative *HR/SCL* indices reaching up to 50%, but also reveal quantitative relationships between physical environmental parameters (i.e., pollutant concentrations and noise levels) and psychophysiological responses through in-situ measurements.

These findings complement previous studies, including the work of Zhou et al. (2026) on the restorative benefits of soundscapes in residential environments and the systematic review by Zhang et al. (2026) on the effects of music interventions on heart rate variability. While the latter confirmed the regulatory influence of music on the autonomic nervous system, most existing evidence has been derived from laboratory or otherwise controlled settings. By contrast, the present study demonstrates the feasibility and effectiveness of piano soundscapes as a low-intervention strategy under realistic environmental conditions where pollutant exposure cannot be eliminated. The results provide new empirical evidence for the fields of urban environmental psychology and soundscape design, highlighting the potential of music-based soundscape interventions to enhance human well-being in polluted urban environments.

### 4.3 The limitation of the present study

This study was conducted through field measurements. Although two comparative testing conditions (i.e., with and without piano performance) were implemented, slight differences in air pollution levels and noise exposure remained between the two testing days, which may have introduced some uncertainty into the experimental results. Future work will involve more controlled laboratory-scale experiments based on established methodologies in the existing literature to improve measurement accuracy (Foellmer and Tyler, 2025). This study focused solely on the effects of traffic-related air pollution and noise on individuals’ physiological responses and subjective perceptions, without accounting for the potential interactions with outdoor thermal comfort and wind sensations. In reality, thermal discomfort and traffic-related pollution/noise may exert complex combined stress effects on individuals. Future research will therefore include more systematic field measurements and laboratory-scale experiments to investigate these interactions. In addition, this study did not consider the influence of different types of piano performance on physiological responses, nor did it incorporate a broader range of physiological indicators (e.g., electroencephalography signals). Future studies will conduct more comprehensive field investigations to account for a wider set of influencing factors.

In this study, all volunteers were healthy males aged approximately 25–30 years. Therefore, the findings may be primarily applicable to similar outdoor leisure settings and healthy young adults, which constitutes a limitation of the present work. Future studies will investigate the influence of different outdoor spatial environments (e.g., pedestrian streets, urban parks, and building undercroft spaces), temporal characteristics (e.g., seasons and public holidays), and participant characteristics (e.g., age, gender, health status, and educational background) on the effectiveness of piano performance interventions. In addition, the impact of cultural differences across cities should be carefully considered in future research, as the level of public acceptance and appreciation of piano performances may vary considerably among different urban contexts.

The acceptance and perception of piano performances may vary substantially among different population groups, depending on factors such as city of residence, gender, age, health status, educational background, and occupation. Owing to individual differences, some participants may even perceive the influence of piano performances as more pronounced than the adverse effects of traffic noise. Therefore, future studies will comprehensively consider these individual-specific factors and conduct more extensive experimental measurements and subjective surveys to further investigate the heterogeneous effects of piano performance interventions on human perception and physiological responses.

## 5 Conclusion

The present study employed in-situ field measurements to investigate the mitigating effects of outdoor piano performance on individuals’ psychological responses to traffic-related air pollution and noise. The current study further analyzed the quantitative relationships between physical and psychological parameters, and complemented these findings with a subjective questionnaire survey. Based on the results described above, some critical conclusions can be drawn as follows:

(1) The air pollution and noise induced by road traffic have obviously affected human psychological reactions. Once the air pollutant concentration (NO_2_, PM_10_) and noise level increase, the relative *HR*/*SCL* indices are higher;
(2) The presence of outdoor piano performance is capable of mitigating psychological reactions caused by traffic emissions, and the relative *HR*/*SCL* indexes can be reduced up to 50%, significant inter-departmental differences are found in HR/SCL indices (Wilcoxon rank-sum test) for the scenarios with and without piano performance.
(3) The temporal variation patterns of the *HR/SCL* index were generally consistent across volunteers throughout the day, while the magnitude of *HR/SCL* index fluctuations at specific time points could exceed 1%.
(4) The vehicular exhaust is barely perceptible to the participants, while traffic noise is clearly perceived by all volunteers and elicited varying degrees of dissatisfaction.

## Data Availability

The datasets presented in this study can be found in online repositories. The names of the repository/repositories and accession number(s) can be found in the article/supplementary material.
